# A Study of Orthopedic Patient Leaflets and Readability of AI-Generated Text in Foot and Ankle Surgery (SOLE-AI)

**DOI:** 10.7759/cureus.75826

**Published:** 2024-12-16

**Authors:** Alexander Jaques, Karim Abdelghafour, Oliver Perkins, Helen Nuttall, Omar Haidar, Karanjeev Johal

**Affiliations:** 1 Trauma and Orthopedics, Lister Hospital, Stevenage, GBR; 2 Trauma and Orthopedics, Cairo University Hospitals, Cairo, EGY; 3 Vascular Surgery, Lister Hospital, Stevenage, GBR; 4 General Surgery, Imperial College Healthcare NHS Trust, London, GBR

**Keywords:** artificial intelligence (ai), elective foot and ankle surgery, orthopaedics surgery, patient information leaflet, readability analysis

## Abstract

Introduction

The internet age has broadened the horizons of modern medicine, and the ever-increasing scope of artificial intelligence (AI) has made information about healthcare, common pathologies, and available treatment options much more accessible to the wider population. Patient autonomy relies on clear, accurate, and user-friendly information to give informed consent to an intervention. Our paper aims to outline the quality, readability, and accuracy of readily available information produced by AI relating to common foot and ankle procedures.

Materials and methods

A retrospective qualitative analysis of procedure-specific information relating to three common foot and ankle orthopedic procedures: ankle arthroscopy, ankle arthrodesis/fusion, and a gastrocnemius lengthening procedure was undertaken. Patient information leaflets (PILs) created by The British Orthopaedic Foot and Ankle Society (BOFAS) were compared to ChatGPT responses for readability, quality, and accuracy of information. Four language tools were used to assess readability: the Flesch-Kincaid reading ease (FKRE) score, the Flesch-Kincaid grade level (FKGL), the Gunning fog score (GFS), and the simple measure of gobbledygook (SMOG) index. Quality and accuracy were determined by using the DISCERN tool by five independent assessors.

Results

PILs produced by AI had significantly lower FKRE scores when compared to BOFAS -40.4 (SD: ±7.69) compared to 91.9 (SD: ±2.24) (p ≤ 0.0001), indicating poor readability of AI-generated text. DISCERN scoring highlighted a statistically significant improvement in accuracy and quality of human-generated information across two PILs with a mean score of 55.06 compared to 46.8. FKGL scoring indicated that the required grade of students to understand AI responses was consistently higher than compared to information leaflets at 11.7 versus 1.1 (p ≤ 0.0001). The number of years spent in education required to understand the ChatGPT-produced PILs was significantly higher in both GFS (14.46 vs. 2.0 years) (p < 0.0001) and SMOG (11.0 vs. 3.06 years) (p < 0.0001).

Conclusion

Despite significant advances in the implementation of AI in surgery, AI-generated PILs for common foot and ankle surgical procedures currently lack sufficient quality, depth, and readability - this risks leaving patients misinformed regarding upcoming procedures. We conclude that information from trusted professional bodies should be used to complement a clinical consultation, as there currently lacks sufficient evidence to support the routine implementation of AI-generated information into the consent process.

## Introduction

The advent of artificial intelligence (AI)-driven software has given patients new means through which to find information about healthcare pathologies and procedures. ChatGPT is a novel large language model (LLM) capable of generating text and has an impressive ability to engage in human-like conversation [1]. Artificial intelligence has shown real promise with respect to the potential scope and implementation in healthcare in an evolving information age. The use of ChatGPT has grown in the fields of medical education and research and, more recently, foot and ankle surgery [2-4].

Despite such advances since its development, questions remain regarding the validity of AI-generated content, especially when compared to traditional, clinician-authored patient information. The quality of AI-generated information is often inconsistent and unreliable. It's essential that patients receive clear, accurate, and comprehensive details, especially when faced with significant medical decisions like surgical interventions. Ensuring access to high-quality educational materials is not just important - it is absolutely critical for making informed, confident decisions about their health.

The Study of Orthopedic Patient Leaflets and Readability of AI-Generated Text in Foot and Ankle Surgery (SOLE-AI) study aims to conduct a retrospective analysis comparing patient information material created by the British Orthopaedic Foot and Ankle Surgery (BOFAS), a reputable specialist society, with those created by ChatGPT (version 3.5). The study will look at three common surgical procedures and compare quality, readability, reliability, and accuracy.

This analysis will provide insight into whether AI-generated patient education materials can be considered a viable alternative to human-authored patient information leaflets (PILs) and whether they meet the standards required for informed decision-making in a surgical setting. Ultimately, it will highlight the strengths and limitations of using AI for patient information.

## Materials and methods

A retrospective analysis and qualitative assessment of patient information materials comparing the quality and readability of AI-generated text and human writing.

The BOFAS is a subspecialist society of the British Orthopaedic Society (BOA) and a UK-registered charity (charity number: 326114) and has authored several PILs aimed to supplement the consenting consultation ahead of surgical intervention. Leaflets for ankle arthroscopy [5], hindfoot fusion [6], and gastrocnemius release surgery [7] were accessed from the BOFAS website. Texts were compared against responses by ChatGPT, the artificial intelligence LLM developed by OpenAI using the GPT-3.5 framework (Introducing ChatGPT 2023). The ChatGPT prompt was "I am a patient who has been offered (Foot and Ankle Orthopaedic Procedure). What do I need to know before agreeing to have this procedure?". ChatGPT inputs and responses are detailed in the appendices below (Appendices A-C).

The text was copied from both the PILs and ChatGPT responses and scored using various scoring indexes for quality, accuracy, and readability.

Quality and accuracy assessments were performed by five independent assessors using the DISCERN system, a tool used to qualitatively assess healthcare treatments. Four unique language scoring systems, derived from the WebFX online readability test, were used to assess readability: the Flesch-Kincaid reading ease (FKRE) score, the Gunning fog score (GFS), the Flesch-Kincaid grade level (FKGL), and the simple measure of gobbledygook (SMOG) index [8]. The FKRE formula produces a numerical result on a scale from 0 to 100 with readability and increasing scores. SMOG and GFS indices quantify the number of years in education required to read and understand a passage of text, with lower scores indicating improved levels of readability.

Inter-assessor reliability was measured using Cohen’s Kappa Statistic. The Kolmogorov-Smirnov test was used to assess the normality of the data (Appendix D). Statistical analysis was performed in Microsoft Excel (Microsoft Corp., Redmond, USA).

## Results

ChatGPT and BOFAS responses were assessed for readability and quality using four distinct readability scoring tools and the DISCERN tool, respectively. PILs produced by BOFAS attained higher mean scores in FKRE (91.933 vs. 40.433) (p < 0.001) when compared to AI-generated text.

Table 1 details that the expected grade of students required to understand the information provided was significantly lower for BOFAS leaflets at 1.1 ± 0.4 compared to 11.7 ± 1.13 (p < 0.001) (Table 1).

**Table 1:** Readability metric scoring of ChatGPT and BOFAS patient information leaflets FKRE: Flesch-Kincaid reading ease; FKGL: Flesch-Kincaid grade level; GFS: Gunning fog score; SMOG: simple measure of gobbledygook; GRS: gastrocnemius release surgery; AA: ankle arthroscopy; HF: hindfoot fusion surgery

Readability scores	ChatGPT				BOFAS			
	GRS	AA	HF	Mean	GRS	AA	HF	Mean
FKRE	37.8	34.4	49.1	40.433	91.4	90	94.4	91.933
FKGL	12.4	12.3	10.4	11.7	1.1	1.5	0.7	1.1
GFS	14.5	15.4	13.5	14.467	1.8	2.3	2	2.0333
SMOG	11.3	11.5	10.3	11.033	3.1	3.3	2.8	3.0667

The Gunning-Fog score and simple measure of gobbledygook analysis determine the number of years spent in education that is required to fully understand a passage of text. Both metrics demonstrated a strong statistically significant difference, with GFS analysis indicating 14.46 ± 0.95 years were required to understand ChatGPT responses compared with 2.03 ± 0.25 years for BOFAS (p < 0.001). Human-generated text scored significantly lower on SMOG indexing, estimating 3.06 ± 0.25 years versus 11.03 ± 0.64 years required (p < 0.001).

All four readability analysis metrics suggest improved readability of clinician-generated texts.

The DISCERN tool was used to assess the accuracy and reliability of the information provided. As demonstrated in Table 2, below, BOFAS PILs produced higher DISCERN scores across all three selected procedures. Cronbach's alpha analysis was performed to assess the inter-assessor reliability of DISCERN scoring, demonstrating excellent reliability between five independent assessors with a score of 0.9267.

**Table 2:** Comparing DISCERN tool scoring for ChatGPT and BOFAS leaflets HF: Hindfoot fusion surgery, AA: ankle arthroscopy, GRS: gastrocnemius release surgery * signifies a p-value < 0.05; ** signifies a p-value < 0.001

DISCERN tool score	ChatGPT				BOFAS			
	HF	AA	GRS	Mean	HF	AA	GRS	Mean
	46.2	47	47.2	46.8	55	59.4*	50.8**	55.067

There was a statistically significant difference seen in the DISCERN scores for ankle arthroscopy, with BOFAS scoring 59.4 versus 47 (p < 0.05) and gastrocnemius release surgery, 50.8 versus 46.8 (p < 0.001).

## Discussion

Over the past two decades, the use of online resources has increased to the point where it is now considered a standard practice. It has been shown that 84.9% of patients visiting orthopedic clinics access the Internet, and 64.7% of patients utilize online platforms to obtain information about their conditions [9].

AI chatbots have been used to deliver medical advice and patient information since the 1990s. Increased interest in recent years has been attributed to more readily available online sources with the success of the AI chatbots in providing medical advice. AI can help selective patients in decision-making and in managing their’ expectations [10]. ChatGPT is useful for bridging the communication gap between patients and surgeons; it can provide complex medical information in simple terms, allowing for improved understanding of complex surgical procedures, alleviating patient anxiety, and helping in decision-making [11].

We report in our study significantly improved readability of human-generated information when processed through four unique metrics. LLM Chatbot text was associated with a statistically significant increase in the number of years in education required to understand the information provided. DISCERN scoring also outlined improved accuracy and reliability of procedure-specific information.

Multiple studies have reported the use of AI chatbots. Sparks et al. evaluated the precision of ChatGPT's responses to patients' inquiries about 40 orthopedic conditions, contrasting them with data from the American Academy of Orthopaedic Surgeons (AAOS) Orthoinfo website [12]. ChatGPT provided significantly fewer treatment options and risk factors but a comparable number of symptoms. Surgical treatment descriptions were often vague. The study concluded that while ChatGPT provides at least moderately accurate orthopedic information, it lacks comprehensive details on treatment and risk factors, making professional resources like AAOS more reliable for patient information.

Studies examining the accuracy of ChatGPT's information in conditions such as shoulder impingement and hip osteoarthritis found similar results, concluding that ChatGPT occasionally provided controversial risk factors, lacked information about treatment options, was largely superficial, and occasionally missed important up-to-date research findings [13,14]. The use of AI in foot and ankle surgery has been reported in multiple studies, assessing the imaging analysis for some conditions such as ankle fractures, Lisfranc injuries, Achilles tendinitis, and calcaneal fractures [15-18].

AI was found to be an excellent information source for patient education and surgeons interested in foot and ankle surgery in a systematic review looking at 31 studies related to the use of AI in foot and ankle surgery [19]. In a study assessing the quality and educational benefit of ChatGPT responses to common foot and ankle conditions, the educational value of the AI-generated responses was variable; 4.5% of responses had a bottom-tier rating, 27.3% of responses had a middle-tier rating, and 68.2% of responses had a top-tier rating after assessment by two independent reviewers [20].

ChatGPT is limited in its current scope due to the way in which it acquires information from various online resources, with some being unreliable or incorrect [21].

An element that might limit the use of ChatGPT is that some professional bodies, such as the Royal College of Surgeons (RCS), state in their guidelines that patient leaflets should be provided to patients where possible to aid decision-making, where leaflets from professional bodies should be the main source of patient education, with no guidance regarding the use of AI in patient information [22]. Recent studies in various surgical fields have found AI-produced information to be of adequate quality and accuracy; however, our study found poor readability and accuracy [23,24]. This discrepancy could potentially be attributed to the varying data availability and nuance of different surgical specialties, which could impact the information provided by AI models.

Limitations

There were several limitations to this study, including the use of one resource (BOFAS) to compare against the ChatGPT responses. First, engagement with the material provided relies on patients placing trust in these scientific bodies as a primary source for accurate information. Second, ChatGPT was prompted with one inquiry, which was, “I am a patient who has been offered (foot and ankle orthopedic procedure). What do I need to know before agreeing to have this procedure?” Different phrasing may potentially have resulted in more detailed or structured information [25].

The findings of this study were based on responses from ChatGPT 3.5, which was reported to have less factual and less expansive responses compared with the paid version, GPT 4.0. This study utilized the free version, as it is more practical for most patients to use. Despite the improvements noted from 3.5 to 4.0, we believe that they should not replace information given by trusted and professional bodies at this time [26].

To ensure the scientific validity of information provided by ChatGPT, a systematic and comprehensive evaluation is necessary to assess its accuracy, reliability, and credibility.

## Conclusions

We conclude that ChatGPT can serve as a valuable tool for patient information and education about their conditions. However, the accuracy of this information can occasionally be questioned, underscoring the importance of direct input and review from orthopedic surgeons and orthopedic societies to prevent incomplete or, worse, misinformation of patients. Despite its usefulness, ChatGPT cannot take the place of professional medical expert advice. Clinicians should lead the consenting process to fully inform the patient of the risks and benefits of intervention, and this study reinforces the need for very judicious integration of AI information into clinical practice. Further research is required to assess the variance in AI-generated PILs across both medical and surgical specialties.

## Appendices

Appendix A

ChatGPT Input

I am a patient who has been offered hindfoot fusion surgery. What do I need to know before agreeing to have this procedure?

ChatGPT Response

Ankle fusion (also known as ankle arthrodesis) is a surgical procedure that involves permanently joining the bones of your ankle to relieve pain, usually caused by severe arthritis, trauma, or deformity. Before agreeing to this procedure, it’s important to understand the following key points:

1. Purpose of the Surgery

The goal of ankle fusion is to relieve pain and improve function by eliminating motion in the damaged joint, which can be very painful in cases of arthritis or injury. It is often recommended when other treatments (physical therapy, medications, or even less invasive surgeries) have failed to provide relief.

2. Procedure Overview

During the surgery, the damaged cartilage is removed, and the bones of the ankle (tibia and talus) are fused together using screws, plates, or rods. Over time, the bones heal and grow together into one solid unit.

3. Recovery

Healing time: Full recovery can take anywhere from 6 to 12 months, depending on your health, age, and how well you follow post-op instructions. Mobility: The fused ankle will no longer have the ability to move up and down, but side-to-side motion from other joints in the foot remains. This could slightly alter your walking gait, but many patients adapt well over time. Non-weight bearing: You’ll likely need to avoid putting weight on the ankle for several weeks or months, which may require crutches or a wheelchair initially.

4. Outcomes

Pain relief: The surgery is highly effective for reducing or eliminating ankle pain due to arthritis or injury. Functionality: While the ankle will be stiff after fusion, most people find they can walk, climb stairs, and engage in many normal activities without significant limitations. However, high-impact activities like running may be more challenging. Long-term success: Ankle fusion generally has good long-term success rates, but it may place extra stress on other joints (such as the knee or hip), which could potentially cause problems in the future.

5. Risks and Complications

Infection: As with any surgery, there’s a risk of infection, which may require additional treatment. Non-union: Sometimes the bones don’t heal properly, leading to a condition called non-union. This might require another surgery. Changes in walking: Because of the lack of ankle movement, your walking pattern may change, which could lead to issues in other parts of your body, like the knees or lower back. Hardware issues: Screws or plates can sometimes cause discomfort and might need to be removed.

6. Alternatives

Ankle replacement: In some cases, a total ankle replacement may be an option, where the ankle joint is replaced with an artificial joint. This preserves some motion in the joint, but it is not suitable for all patients, particularly those with severe deformities or young, active individuals. Bracing or orthotics: Some patients manage pain and functional limitations with special braces or orthotics, but this may only provide temporary relief.

7. Lifestyle Impact

You may need to make adjustments to your daily activities, especially during the recovery period. Discuss with your surgeon how the procedure will affect your lifestyle, including work, exercise, and long-term mobility. It’s essential to have a detailed discussion with your surgeon about the risks, benefits, and alternatives based on your specific situation.

Appendix B

ChatGPT Input

I am a patient who has been offered Gastrocnemius Release surgery. What do I need to know before agreeing to have this procedure?

ChatGPT Response

A gastrocnemius release (also known as a gastrocnemius recession or calf muscle lengthening) is a surgical procedure that is performed to relieve tightness in the calf muscle (gastrocnemius) and improve flexibility in the ankle. It is often recommended for patients with conditions like equinus deformity, plantar fasciitis, or Achilles tendon issues, where calf muscle tightness limits movement and causes pain.

Here are some key points to consider before agreeing to the procedure:

1. Purpose of the Surgery

Relieve calf tightness: The gastrocnemius muscle can become overly tight, limiting how far your ankle can flex upwards (dorsiflexion). This can cause foot pain, walking difficulties, and increase strain on the Achilles tendon or plantar fascia. Improve ankle mobility: By releasing part of the muscle, the surgery aims to increase ankle flexibility and reduce stress on other parts of the foot and lower leg.

2. Conditions That May Benefit

Plantar fasciitis: Tight calf muscles can worsen plantar fasciitis by increasing pressure on the plantar fascia (the ligament running along the bottom of your foot). Achilles tendinitis: Overstrain of the Achilles tendon can occur due to reduced ankle flexibility, often due to a tight gastrocnemius. Flatfoot or high arches: This procedure can help relieve the stress associated with abnormal foot postures. Equinus deformity: This condition involves limited upward bending of the ankle, and gastrocnemius release can help correct this.

3. Procedure Overview

The surgery involves releasing or lengthening the gastrocnemius muscle. This can be done through a small incision behind the knee or at the lower part of the calf. The surgeon will either cut the tendon of the gastrocnemius or make a small cut in the muscle itself to lengthen it and reduce tightness.

4. Recovery

Healing time: Recovery typically takes about 4 to 6 weeks, but it can take up to 3 months for a full return to normal activities. Weight-bearing: Some patients can bear weight on the foot immediately after surgery, while others may need crutches or a walking boot for a few weeks, depending on the surgical approach. Physical therapy: Rehabilitation will include stretching and strengthening exercises to help restore normal mobility and prevent tightness from recurring.

5. Outcomes

Increased ankle flexibility: Most patients experience improved ankle range of motion and relief from pain caused by tight calf muscles. Improved foot function: Relieving tension in the calf can help resolve foot pain, improve walking mechanics, and reduce stress on the Achilles tendon or plantar fascia. Long-term benefits: The procedure generally has good long-term results, though maintaining flexibility with regular stretching exercises is essential to prevent tightness from returning.

6. Risks and Complications

Over-lengthening: There's a risk of lengthening the muscle too much, which could result in weakness or difficulty pushing off during walking. Nerve injury: In rare cases, nerves in the area could be damaged during the procedure, leading to numbness or weakness. Scarring: Some patients may experience scarring at the incision site, though this is usually minimal. Recurrence of tightness: Without proper rehabilitation, tightness can return over time.

7. Alternatives

Physical therapy: Stretching and strengthening exercises under the guidance of a physical therapist can sometimes improve calf flexibility without surgery. Bracing or orthotics: In some cases, special orthotics or braces can reduce symptoms, though they don’t directly address the underlying calf tightness. Botox injections: Injections of botulinum toxin (Botox) into the calf muscle can temporarily reduce tightness, but the effects are short-term and may not be a permanent solution.

8. Impact on Lifestyle

After the surgery, you may need to temporarily limit weight-bearing activities and follow a rehabilitation plan. Long-term, you should be able to return to most normal activities with improved comfort and mobility. It’s important to discuss your specific symptoms and goals with your surgeon, as this procedure is generally elective and designed to address a quality-of-life issue. 

Appendix C

ChatGPT Input

I am a patient who has been offered an ankle arthroscopy. What do I need to know before agreeing to have this procedure?

ChatGPT Response

Ankle arthroscopy is a minimally invasive surgical procedure used to diagnose and treat problems inside the ankle joint. Before deciding to go ahead with this procedure, here are some key points you should know:

1. Purpose of the Surgery

Diagnosis and treatment: Ankle arthroscopy allows the surgeon to view the inside of your ankle joint with a small camera (arthroscope) and perform repairs through small incisions. It can help treat conditions like: Torn ligaments or tendons, Bone spurs or loose fragments, Arthritis, Impingement (pinching of soft tissues), inflammation in the joint lining (synovitis). Minimally invasive: The procedure is less invasive than open surgery, leading to faster recovery times.

2. Procedure Overview

The surgeon makes small incisions around the ankle and inserts the arthroscope to visualize the joint. Using additional small instruments, they can remove debris, repair tissues, or correct structural issues. Ankle arthroscopy is usually performed under general or local anesthesia, depending on your surgeon's recommendation and the complexity of the procedure.

3. Recovery

Faster healing: Compared to open surgery, recovery is typically quicker, though the exact timeline depends on the extent of the damage repaired. Activity restrictions: You may need to avoid weight-bearing activities for a few days or weeks, depending on the procedure. Crutches or a walking boot might be necessary during the early stages of healing. Physical therapy: Rehabilitation exercises are often recommended to restore range of motion, strength, and flexibility. Completing physical therapy is important to ensure a full recovery. Return to normal activities: Many patients can return to normal activities within 4-6 weeks, though this may vary.

4. Outcomes

Pain relief: The procedure is effective for reducing or eliminating pain caused by certain conditions such as bone spurs, arthritis, or ligament injuries. Improved mobility: Most patients experience improved ankle function and range of motion, though this depends on the condition being treated. Return to activity: Many patients can resume physical activities, including sports, after a successful arthroscopy and rehabilitation.

5. Risks and Complications

Infection: As with any surgery, there is a small risk of infection. Nerve or blood vessel damage: There is a risk, though rare, of damage to nearby nerves or blood vessels. Stiffness or swelling: Some patients experience stiffness or swelling post-surgery, but these typically resolve with time and physical therapy. Blood clots: Though uncommon, blood clots (deep vein thrombosis) can occur after surgery.

6. Alternatives

Non-surgical options: Before opting for surgery, it’s essential to consider non-invasive treatments like physical therapy, orthotics, injections (e.g., cortisone), or medications for pain and inflammation. Open surgery: For more complex ankle conditions, open surgery might be necessary instead of or in addition to arthroscopy.

7. Long-Term Impact

After successful recovery, many patients can return to normal or even high-impact activities like sports. However, the extent of your recovery may depend on the severity of the original condition, as well as your adherence to post-op care and rehabilitation. Discuss these points with your surgeon to ensure you understand the procedure’s potential benefits, risks, and what you can expect in terms of recovery.

Appendix D

**Table 3:** Kolmogorov-Smirnov test of normality KS: Kolmogorov-Smirnov test; (D): lower numbers indicating greater Gaussian distribution; FKRE: Flesch-Kincaid reading ease; FKGL: Flesch-Kincaid grade level; GFS: Gunning fog score; SMOG: simple measure of gobbledygook

Readability scores	Mean	Median	Standard deviation	KD (D)	Gaussian distribution
FKRE	66.1833333	69.55	28.6598267	0.31868	Yes
FKGL	6.4	5.95	5.85491247	0.32037	Yes
GFS	8.25	7.9	6.83834775	0.32974	Yes
SMOG	7.05	6.8	4.38531641	0.32555	Yes
